# Minimal association between Th1-specific responses to COVID-19 vaccines and SARS-CoV-2 breakthrough infections in multiple sclerosis patients receiving disease-modifying therapies

**DOI:** 10.3389/fimmu.2025.1682049

**Published:** 2025-10-30

**Authors:** Alessandra Aiello, Assunta Navarra, Shalom Haggiag, Serena Ruggieri, Gilda Cuzzi, Valentina Vanini, Andrea Salmi, Stefania Notari, Anna Maria Gerarda Altera, Silvia Meschi, Francesca Colavita, Eleonora Cimini, Carla Tortorella, Luca Prosperini, Maria Esmeralda Quartuccio, Simonetta Galgani, Vincenzo Puro, Fabrizio Maggi, Alba Grifoni, Alessandro Sette, Emanuele Nicastri, Claudio Gasperini, Delia Goletti

**Affiliations:** ^1^ Translational Research Unit, National Institute for Infectious Diseases “Lazzaro Spallanzani” Istituto di Ricovero e Cura a Carattere Scientifico (IRCCS), Rome, Italy; ^2^ Clinical Epidemiology Unit, National Institute for Infectious Diseases “Lazzaro Spallanzani” Istituto di Ricovero e Cura a Carattere Scientifico (IRCCS), Rome, Italy; ^3^ Department of Neurosciences, Azienda Ospedaliera San Camillo Forlanini, Rome, Italy; ^4^ Unità Operativa Semplice (UOS) Professioni Sanitarie Tecniche, National Institute for Infectious Diseases “Lazzaro Spallanzani” Istituto di Ricovero e Cura a Carattere Scientifico (IRCCS), Rome, Italy; ^5^ Cellular Immunology and Pharmacology Laboratory, National Institute for Infectious Diseases “Lazzaro Spallanzani” Istituto di Ricovero e Cura a Carattere Scientifico (IRCCS), Rome, Italy; ^6^ Laboratory of Virology, National Institute for Infectious Diseases “Lazzaro Spallanzani” Istituto di Ricovero e Cura a Carattere Scientifico (IRCCS), Rome, Italy; ^7^ U.O.C. Risk Management, National Institute for Infectious Diseases “Lazzaro Spallanzani” Istituto di Ricovero e Cura a Carattere Scientifico (IRCCS), Rome, Italy; ^8^ Center for Vaccine Innovation, La Jolla Institute for Immunology (LJI), La Jolla, CA, United States; ^9^ Department of Medicine, Division of Infectious Diseases and Global Public Health, University of California San Diego (UCSD), La Jolla, CA, United States; ^10^ Clinical Division of Infectious Diseases, National Institute for Infectious Diseases “Lazzaro Spallanzani” Istituto di Ricovero e Cura a Carattere Scientifico (IRCCS), Rome, Italy

**Keywords:** multiple sclerosis, Th1 cytokines, mRNA vaccines, SARS-CoV-2 infection, T-cell response, disease-modifying therapies

## Abstract

**Background:**

The COVID-19 pandemic highlighted challenges in managing patients with multiple sclerosis (PwMS), as disease-modifying therapies (DMTs) can interfere with immune responses to infections and vaccines.

**Objective:**

This study investigates the spike-specific T-cell response after the third dose of mRNA COVID-19 vaccines in PwMS undergoing DMTs, evaluating different cytokines, beyond IFN-γ, and exploring their potential association with SARS-CoV-2 breakthrough infections (BI).

**Methods:**

We prospectively enrolled 31 PwMS and 27 healthcare workers (HCWs). The spike-specific T-cell response was evaluated by measuring Th1 cytokines (IFN-γ, IL-2, TNF-α) and IP-10 using an easy-to-use whole-blood assay.

**Results:**

Most PwMS mounted a Wuhan spike-specific T-cell response by releasing Th1 cytokines (IFN-γ, IL-2, TNF-α) and IP-10, albeit with significantly reduced Th1 cytokine levels compared to HCWs. Fingolimod-treated patients showed the weakest response with significantly reduced IFN-γ and IL-2 levels compared to HCWs (both p<0.0001), as well as to ocrelizumab (p=0.0018 and p=0.0002, respectively) and cladribine/IFN-β-treated patients (p=0.041 and p<0.0001, respectively). Moreover, a cell-mediated response was observed against the Delta spike variant, and all cytokines correlated with each other. BI occurred in 38.7% of PwMS, with predominantly mild COVID-19 cases. Male sex (IRR: 4.05, p=0.017) and primary progressive MS (IRR: 3.65, p=0.052) were associated with a higher BI incidence rate. Spike-specific T-cell response did not associate with a higher protection against BI.

**Conclusions:**

This study provides an in-depth immunological characterization of the spike-specific T-cell response in PwMS under DMTs, evaluating immunological biomarkers whose relevance may extend beyond COVID-19 for studying immune responses to other infections and vaccinations.

## 1 Introduction

Multiple sclerosis (MS) is an immune-mediated disease that affects the central nervous system, causing demyelination (1). A major breakthrough in the management of MS has been the advent of disease-modifying therapies (DMTs), such as ocrelizumab, fingolimod, cladribine, and interferon (IFN)-β. These therapies target the immune system at different levels, thus potentially compromising the immune response to both infections and vaccinations (2–4).

Consequently, during COronaVIrus Disease 2019 (COVID-19) pandemic, the management of patients with MS (PwMS) raised significant concerns (5). Key issues included the potential increased susceptibility to SARS-CoV-2 infection and the risk of severe COVID-19 outcomes, which vary depending on the DMT used (6, 7). In particular, a more severe outcome of COVID-19 has been reported in PwMS treated with anti-CD20 therapies (8, 9).

The vaccination campaign launched in early 2021 was an effective measure to mitigate the public health impact of COVID-19 by reducing the severity of the disease and hospitalization rates (10–12). Although vaccination against COVID-19 has proven effective, breakthrough infections (BI) have continued to occur due to the progressive weakening of vaccine-induced immunity (13–16) and the emergence of SARS-CoV-2 variants, likely favored by the viral replication in immunocompromised subjects, who are more susceptible to developing persistent infections (17).

However, vaccination continues to be the primary defense against COVID-19 in vulnerable individuals such as PwMS. Several studies, including ours, have demonstrated the immunogenicity of anti-SARS-CoV-2 vaccines in both healthy individuals (18–21) and PwMS (13, 22–27). The collective evidence indicates that most PwMS develop humoral and/or IFN-γ-specific T-cell responses to SARS-CoV-2 spike peptides. Nevertheless, the overall magnitude of these immune responses is reduced in PwMS compared with healthy individuals, and varies depending on the DMTs administered (13, 28–30). Specifically, fingolimod, a sphingosine-1 phosphate receptor modulator, predominantly impairs IFN-γ-specific T-cell response (31–33), while the B-cell-depleting anti-CD20 monoclonal antibody, ocrelizumab, is mostly associated with reduced anti-receptor binding domain (RBD) and neutralizing antibody production after COVID-19 vaccination (34–37). Moreover, the presence of both antibody and cell-mediated immune responses has been associated with a more rapid swab negativization in PwMS; indeed fingolimod-treated patients, who have a compromised IFN-γ-specific T-cell response, tend to require more time to achieve swab-negative status (38).

These results underscore the importance of studying the immune response to SARS-CoV-2 in PwMS, with a focus on the T-cell response (39, 40). In particular, T helper 1 (Th1) lymphocytes are known to play a fundamental role in the immune response against viral infections through the release of key cytokines such as IFN-γ, interleukin (IL)-2, and tumor necrosis factor (TNF)-α, also known as Th1 cytokines (41, 42). IP-10/CXCL-10 (Interferon gamma-induced protein 10) is a chemokine induced by IFN-γ, which plays a pivotal role in the activation and chemoattraction of immune cells, especially T cells, to the sites of inflammation (43).

To date, most studies have primarily assessed the spike-specific T-cell response in terms of IFN-γ production (13, 18, 32, 36, 44), whereas some studies have evaluated the functional spike-specific CD4^+^ and CD8^+^ T cell responses by flow cytometry in PwMS after three doses of SARS-CoV-2 vaccines (22, 45–47). To the best of our knowledge, only one study conducted by Al Rahbani (48) evaluated, beyond IFN-γ, the SARS-CoV-2-specific immune cytokine profile in plasma supernatant of PwMS. However, this analysis did not compare the cytokine response with healthy controls nor correlate it with the risk of BI (48).

Our study aims to investigate additional biomarkers for a more complete evaluation of the cell-mediated immune response to COVID-19 vaccination. This study specifically examined the spike-specific immune response following a third dose of mRNA COVID-19 vaccines by assessing Th1 cytokines (IFN-γ, IL-2, and TNF-α) and IP-10 production using an easy-to-use whole-blood assay in PwMS undergoing various DMTs. Additionally, these responses were compared to those observed in healthcare workers (HCWs), alongside an evaluation of the risk of BI. Moreover, demographic and clinical variables were evaluated for their potential impact on cytokine production.

## 2 Materials and methods

### 2.1 Study cohort

This prospective, longitudinal, single-center study included a cohort of patients diagnosed with MS (PwMS) according to the 2017 revisions of McDonald criteria (49), along with an age- and sex-matched control group of healthcare workers (HCWs).

PwMS were recruited from patients regularly followed at the outpatient clinic at the MS Centre of the Department of Neurosciences of San Camillo Forlanini Hospital (Rome, Italy). Eligibility criteria included receiving three doses of COVID-19 mRNA vaccines (BNT162b2 or mRNA-1273) and ongoing treatment with ocrelizumab, fingolimod, cladribine, or IFN-β.

Healthy controls were recruited among HCWs without immune-suppressive conditions who had received three doses of COVID-19 mRNA vaccines at the National Institute for Infectious Diseases (INMI) – Lazzaro Spallanzani (Rome, Italy).

Enrolled PwMS were followed until either a confirmed SARS-CoV-2 infection or the administration of the fourth vaccine dose. Confirmed SARS-CoV-2 BI were classified by severity as mild, moderate, or severe (50). The enrolment began in March 2021 and was completed with the conclusion of the follow-up in December 2022.

Exclusion criteria for both cohorts included HIV infection, age below 18 years, and prior SARS-CoV-2 infection, defined by a positive antigenic or molecular test and/or detectable anti-nucleoprotein antibodies (anti-N IgG) at baseline.

The study was approved by the Ethical Committee of National Institute of Infectious Diseases “L. Spallanzani” (INMI)-IRCCS (approval numbers 319/2021, 443/2021, 297/2021) and performed in accordance with the ethical standards laid down in the 1964 Declaration of Helsinki and its later amendments. All participants signed a written informed consent before their inclusion in the study.

### 2.2 Sample collection

Blood samples from PwMS were collected in BD Vacutainer tubes containing lithium heparin (Becton Dickinson, Florence, Italy, Cat. 367526) 4–6 weeks after the third dose of COVID-19 mRNA vaccines. Samples obtained at the MS Center of San Camillo Forlanini Hospital were transported to INMI and processed within 2 hours of collection. Blood samples from both PwMS and HCWs were handled according to a standardized protocol routinely used (13, 23, 51).

### 2.3 Antibody testing

The enrolled cohort was screened for prior SARS-CoV-2 infection by assessing anti-N-IgG as per the manufacturer’s instructions (Architect^®^ i2000sr, Abbott Diagnostics, Chicago, IL, USA). Anti-N IgG were considered positive when index values, calculated as the ratio of sample (S) to cut-off (CO), were ≥1.4. The anti-SARS-CoV-2 antibody response to COVID-19 vaccination was assessed in terms of anti-receptor-binding domain (RBD) antibodies (anti-RBD Abs) and neutralizing ones. Anti-RBD IgG levels, expressed as binding antibody units (BAU)/mL, were measured according to the manufacturer’s instructions (Architect^®^ i2000sr, Abbott Diagnostics, Chicago, IL, USA), and identify a positive response when ≥7.1 BAU/mL. Neutralising antibodies were assessed by the micro-neutralization assay previously reported (52), using the SARS-CoV-2/Human/ITA/PAVIA10734/2020 (provided by Fausto Baldanti, Pavia, Italy). A neutralizing titre ≥10, corresponding to the first dilution tested, was considered positive.

### 2.4 Spike-specific cell response

A whole blood assay was used to assess the spike-specific T-cell response. Specifically, 600 µL of blood was stimulated in a 48-well plate with peptide pools covering the spike protein sequence derived from SARS-CoV-2 Wuhan-Hu-1 (Wuhan spike) and the Delta variant (Delta spike). The Wuhan spike pool consisted of equal amounts of three peptide pools (PepTivator^®^ Prot_S1, Prot_S, and Prot_S+, Miltenyi Biotec, Bergisch Gladbach, Germany, Cat. 130–127–048, Cat. 130–126–701, and Cat. 130–127–312) used at a final concentration of 0.1 µg/mL. The Delta spike pool, consisting of overlapping 15-mer peptides, was designed based on the GISAID ID: EPI_ISL_2020950, and used at 0.1 μg/mL. To verify the immunocompetence of the enrolled subjects, Staphylococcal enterotoxin B (SEB) (Merck Life Science, Milan, Italy, Cat. S4881) was used at 200 ng/mL. Following overnight incubation, the stimulated plasma was collected and stored at -80 °C until further analysis. Th1 cytokines (IFN-γ, TNF-α, IL-2) and IP-10 were quantified using the ELLA Simple Plex Human Assay (Bio-Techne, Minneapolis, MN, USA, Cat. SPCKC-PS-003978 customized kit). The detection limits for IP-10, IFN-γ, IL-2 and TNF-α were 0.60 pg/mL, 0.17 pg/mL, 0.54 pg/mL, and 0.3 pg/mL, respectively. Data were reported after subtracting the unstimulated value.

### 2.5 Statistical analysis

Statistical analyses were performed using GraphPad Prism software (version 8, Dotmatics, Boston, MA 02110) and Stata (StataCorp. 2021. Stata Statistical Software: Release 17. TX: StataCorp LLC, College Station, TX, USA). Categorical variables were reported as absolute values and relative percentages, whereas the continuous ones were expressed as medians and interquartile ranges (IQR). Mann-Whitney U and Kruskal-Wallis tests, followed by Dunn’s multiple comparisons test, were performed for pairwise and multiple comparisons, respectively. Wilcoxon signed-rank test was used to analyze paired data. For categorical variables, the Fisher’s Exact test was used. Correlations among immunological parameters were assessed by the non-parametric Spearman’s rank test (ρ coefficient).

To account for potential demographic and clinical confounders of cytokine production, a quantile regression analysis was performed. In the analysis, dependent variables (i.e., IFN-γ, IL-2, TNF-α and IP-10) and the following covariates were included: age, sex, body mass index (BMI), type of DMT, disease and treatment duration, lymphocyte count, Expanded Disability Status Scale (EDSS) score, presence of comorbidities, MS disease phenotype, and time from the third vaccine dose to sample collection. Covariates with p < 0.05 were entered in the stepwise regression model to identify the most influential factors.

To determine the incidence rate ratio (IRR) of SARS-CoV-2 infection based on demographic, clinical, and immunological parameters, an univariable Poisson regression model was applied. Two-tailed p values were considered statistically significant if <0.05.

## 3 Results

### 3.1 Characteristics of the enrolled cohort

**Figure 1:**
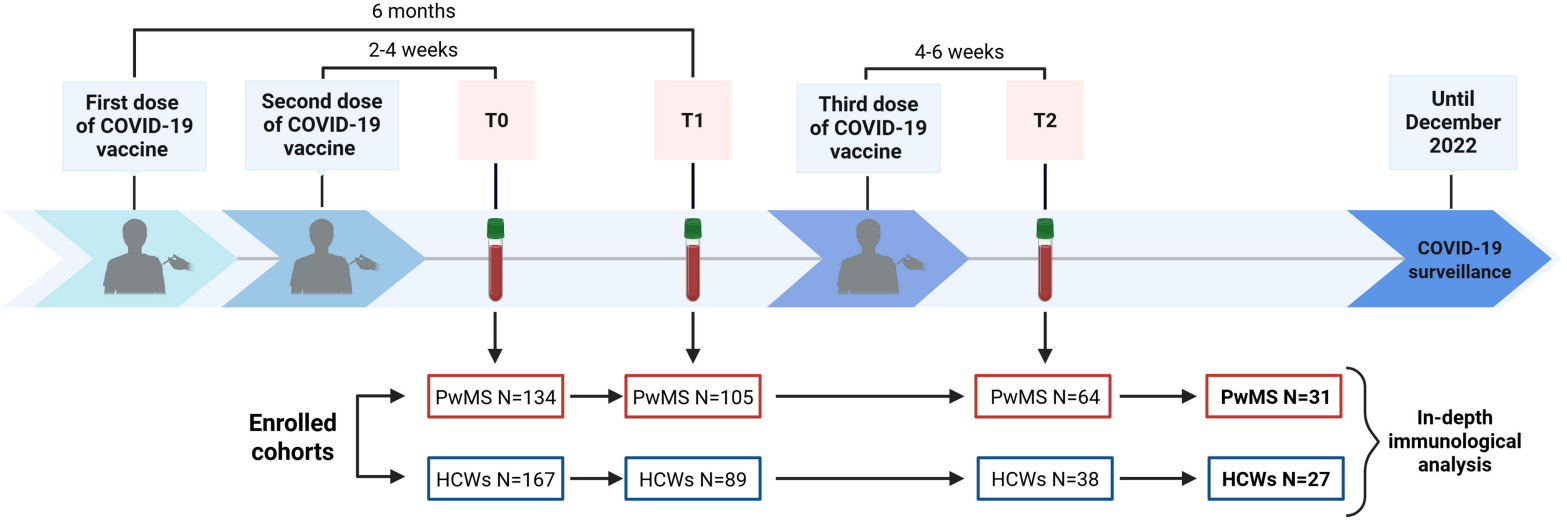
Timeline of COVID-19 vaccination and blood sample collection. The flow chart displays the enrolment and sample collection at T0 (2–4 weeks after the second dose), T1 (6 months after the first dose) and T2 (4–6 weeks after the third dose), excluding subjects lost to follow-up. Out of 64 PwMS and 38 HCWs who completed blood sampling at all designated time points, a convenience sample consisting of 31 PwMS and 27 HCWs was selected for an in-depth immunological characterization. COVID-19, COronaVIrus Disease 2019; HCWs, healthcare workers; PwMS, patients with multiple sclerosis. Created in https://BioRender.com.

From established cohorts of 167 HCWs and 134 PwMS previously evaluated for immune response to COVID-19 vaccination in related studies (13, 23, 38, 53), a subgroup of 27 HCWs and 31 PwMS, who completed blood sampling at all established time points, was selected for an in-depth analysis of the immunological response 4–6 weeks after the third vaccine dose (**Figure 1**).

The characteristics of the study cohort are described in **Table 1**. The two groups were matched for age and sex; both cohorts showed a female predominance exceeding 75%, reflecting the approximately 3:1 female-to-male ratio in MS, and the higher proportion of women among HCWs in Italy. Among the enrolled PwMS, 10 (32.2%) subjects were treated with ocrelizumab, 13 (41.9%) with fingolimod, 3 (9.7%) with cladribine, and 5 (16.1%) with IFN-β. Most PwMS (87.1%) had a relapsing-remitting MS, and the median duration of the disease was 16 years. The median MS treatment duration at the sample collection was 1.8 years (IQR: 1.0–2.3) for ocrelizumab, 7.4 years (IQR: 5.1–8.5) for fingolimod, 1.6 years (IQR: 0.8–1.9) for cladribine and 9.5 years (IQR: 7.6–18.7) for IFN-β. Only a few subjects reported comorbidities such as cardiovascular and metabolic diseases. Lymphocyte counts significantly differed among DMTs (p=0.003), with fingolimod-treated patients showing the lowest counts. Regarding the other blood cell subsets, no significant differences were found among the different DMTs as reported in **Table 1**. The median time from the third vaccine dose to the blood sample collection in PwMS was 48 days (IQR 43-51), with no differences among treatment subgroups.

**Table 1:** Demographic and clinical characteristics of the 58 subjects enrolled after the third dose of COVID-19 vaccination.

Characteristics		PwMS	HCWs	P value
n (%)		31 (53.5)	27 (46.5)	
Age median (IQR)		49 (45-55)	47 (35-54)	0.440*
Female n (%)		24 (77.4)	22 (81.5)	0.755^§^
Origin n (%)	West Europe	31 (100)	27 (100)	
BMI (kg/m^2^), median (IQR)		23.7 (20.8-26.1)	NA	
Presence of comorbidities		5 (16.1)	NA	
MS disease duration (years), median (IQR)		16 (6-22)	–	
MS Course n (%)	Relapsing-remitting	27 (87.1)	–	
	Primary-progressive	4 (12.9)	–	
EDSS score, median (IQR)		3 (1-4.5)	–	
MS treatment duration (years), median (IQR)		4.7 (1.8-8.0)	–	
Multiple Sclerosis Treatment n (%)	Ocrelizumab	10 (32.2)	–	
	Fingolimod	13 (41.9)	–	
	Cladribine	3 (9.7)	–	
	IFN-β	5 (16.2)	–	
Lymphocytes count Median x10^3^/µL (IQR)^#^	Ocrelizumab	1.6 (1.4-1.9)	–	0.003 **
	Fingolimod	0.7 (0.6-1.0)	–	
	Cladribine	0.9 (0.8-1.4)	–	
	IFN-β	1.6 (1.5-1.8)	–	
Neutrophils count Median x10^3^/µL (IQR)^#^	Ocrelizumab	3.5 (2.9-4.6)		0.094 **
	Fingolimod	3.1 (2.4-4.6)		
	Cladribine	2.4 (1.8-2.5)		
	IFN-β	3.9 (2.9-5.2)		
Monocytes count Median x10^3^/µL (IQR)^#^	Ocrelizumab	0.5 (0.4-0.6)		0.178 **
	Fingolimod	0.5 (0.4-0.6)		
	Cladribine	0.3 (0.3-0.4)		
	IFN-β	0.7 (0.5-0.9)		
Eosinophils count Median x10^3^/µL (IQR)^#^	Ocrelizumab	0.16 (0.08-0.24)		0.123 **
	Fingolimod	0.08 (0.02-0.12)		
	Cladribine	0.08 (0.07-0.13)		
	IFN-β	0.09 (0.06-0.11)		
Basophils count Median x10^3^/µL (IQR)^#^	Ocrelizumab	0.04 (0.03-0.06)		0.05 **
	Fingolimod	0.03 (0.01-0.03)		
	Cladribine	0.04 (0.00-0.06)		
	IFN-β	0.01 (0.00-0.02)		
Time from third dose to sample, median days (IQR)		48 (43-51)	31 (29-32)	<0.0001*
Among PwMS with SARS-CoV-2 breakthrough infection				
SARS-CoV-2 breakthrough infection		12 (38.7)	NA	
Days from 3rd dose to infection		135 (100-249)	NA	
Days to negative swab, median (IQR)		13 (10-15)	NA	
COVID-19 severity n (%)	Mild	10 (83.4)	NA	
	Moderate	1 (8.3)	NA	
	Severe	1 (8.3)	NA	
COVID-19 therapy n (%)	Antiviral	3 (27.3)	NA	
	Monoclonal	6 (54.5)	NA	
	Non-steroidal inflammatory drugs	2 (18.2)	NA	

HCWs, healthcare workers; PwMS, patients with multiple sclerosis; BMI, body mass index; EDSS, Expanded Disability Status Scale; IQR, Interquartile range; n, Number; NA, not available.^#^ Blood cell counts available for 28 out of 31 PwMS; * Mann-Whitney U-statistic test; ^§^ Fisher’s Exact test; ** Kruskal-Wallis test.

### 3.2 Profile of cytokines induced by SARS-CoV-2 vaccination

**Figure 2:**
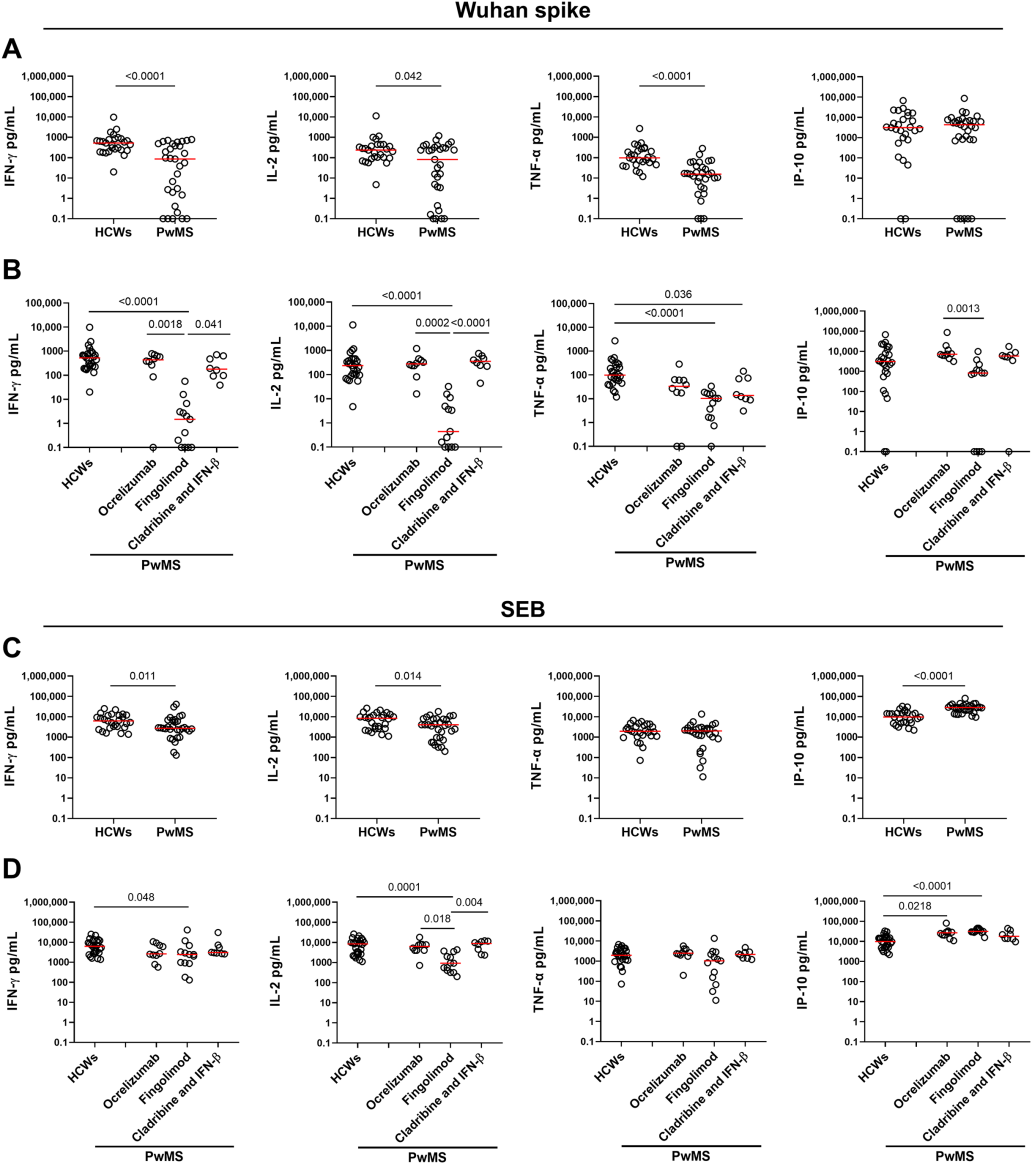
Cytokine/chemokine response to SARS-CoV-2 Wuhan spike peptides and SEB in HCWs and PwMS after 4–6 weeks from the third vaccine dose. Comparison of the cytokine response to Wuhan spike **(A, B)** and SEB **(C, D)** between HCWs and PwMS. **(B, D)** PwMS were stratified by the current therapy into three groups: ocrelizumab (n=10), fingolimod (n=13) and cladribine/IFN-β (n=8). IFN-γ, IL-2, TNF-α and IP-10 concentrations were expressed in pg/mL with median values indicated by red lines. For the statistical analysis, Mann-Whitney U test was performed to compare HCWs and PwMS **(A, C)**, while for the comparison among groups the Kruskal-Wallis test followed by the Dunn’s multiple comparisons test was used **(B, D)**. Differences with p values < 0.05 were considered significant. HCWs, healthcare workers; PwMS, patients with multiple sclerosis; SEB, Staphylococcal enterotoxin B; IFN, interferon; IL, interleukin; TNF, tumor necrosis factor; IP-10, interferon gamma-induced protein 10.

The spike-specific cell response was assessed by measuring Th1 cytokines (IFN-γ, IL-2, and TNF-α) as well as IP-10, an IFN-γ-induced protein, in response to the Wuhan spike peptides. All HCWs responded to Wuhan spike stimulation by producing IFN-γ, IL-2 and TNF-α, while only 25/27 (92.6%) HCWs released IP-10 (**Figure 2A**). Compared with HCWs, PwMS showed a significantly reduced cytokine response to Wuhan spike peptides with a 6-fold decreased IFN-γ level (HCWs median: 525 pg/mL, IQR: 226–852 vs. PwMS median: 86 pg/mL, IQR: 1.5-476.5, p<0.0001) and TNF-α production (HCWs median: 98 pg/mL, IQR: 44.6–270 vs. PwMS median: 15.4 pg/mL, IQR: 3.7-38.7, p<0.0001), and an approximately 3-fold reduction in IL-2 production (HCWs median: 234 pg/mL, IQR: 96–447 vs. PwMS median: 81 pg/mL, IQR: 3.3-325, p=0.042). By contrast, no significant differences were observed for IP-10, between the two groups (**Figure 2A**).

Although most PwMS showed a T-cell specific response, the quantitative response to Wuhan spike significantly varied among DMTs. In particular, patients receiving fingolimod showed an impaired immune response characterized by a significantly reduced production of both IFN-γ, IL-2, and TNF-α compared to HCWs (p<0.0001 for all Th1 cytokines) (**Figure 2B**). The significant differences in IFN-γ and IL-2 production observed between PwMS treated with fingolimod and HCWs persisted even after adjusting for the time interval between the third vaccine dose and sample collection (**Table 2**).

**Table 2:** Comparison between PwMS and HCWs for each cytokine/chemokine in response to the *in vitro* specific stimulation with Wuhan spike-peptides after the third dose of COVID-19 vaccination.

Cytokines/ chemokines	Group	Quantile regression analysis (median)					
		Univariable			Adjusted for time (days) from third vaccine dose to sample collection		
		Coefficient	95% CI	P	Coefficient	95% CI	P
Spike-induced IFN-γ	PwMS_Fing_No vs HCWs	-155	-432; 121	0.265	-140	-504; 225	0.446
	PwMS_Fing_Yes vs HCWs	-523	-830; -216	**0.001**	-516	-906; -126	**0.010**
Spike-induced IL-2	PwMS_Fing_No vs HCWs	74	-67; 214	0.297	93	-82; 269	0.292
	PwMS_Fing_Yes vs HCWs	-234	-389; -78	**0.004**	-207	-394; -19	**0.032**
Spike-induced TNFα	PwMS_Fing_No vs HCWs	-71	-147; 5	0.066	-68	-169; 32	0.177
	PwMS_Fing_Yes vs HCWs	-88	-172; -4	**0.041**	-87	-194; 20	0.111
Spike-induced IP-10	PwMS_Fing_No vs HCWs	3267	-727; 7261	0.107	4214	-1020; 9449	0.112
	PwMS_Fing_Yes vs HCWs	-2298	-6728; 2133	0.303	-1202	-6800; 4397	0.669

PwMS, patients with multiple sclerosis; HCWs, healthcare workers; CI, confidence interval; IFN, interferon; IL, interleukin; TNF, tumor necrosis factor; IP-10, interferon-gamma-induced protein 10. PwMS_Fing_No, are PwMS undergoing ocrelizumab, cladribine and IFN-β. PwMS_Fing_Yes are PwMS undergoing fingolimod. In bold are reported the significant values.

Within the PwMS cohort, fingolimod-treated patients exhibited significantly lower levels of IFN-γ and IL-2 compared to patients treated with ocrelizumab (p=0.0018 and p=0.0002, respectively) or cladribine/IFN-β (p=0.041 and p<0.0001, respectively). Moreover, a significant difference was observed in the IP-10 production between patients receiving ocrelizumab and fingolimod (p=0.0013). In contrast, no significant differences were reported for TNF-α production among PwMS stratified based on DMTs (**Figure 2B**).

To evaluate the impact of demographic and clinical variables on spike-specific responses in PwMS, a quantile regression analysis was conducted. While various covariates were initially identified as potential influencers depending on the cytokine or chemokine assessed, only some variables remained significant following stepwise regression (**Table 3**). The differences in Th1 cytokine and IP-10 levels among patients treated with fingolimod, as compared to those receiving alternative therapies, were validated through stepwise regression analysis (**Table 3**). The MS treatment emerged as the main variable that explained the differences observed in the MS cohort for IFN-γ, IL-2 and IP-10, (p=0.062, p<0.001, p<0.001, respectively). Moreover, the gender was identified as a variable affecting the specific-immune response, with male patients showing higher levels of IFN-γ and TNF-α (p=0.003 and p=0.010) (**Table 3**). Instead, the duration of MS treatment did not appear to influence the cytokine response.

**Table 3:** Quantile and stepwise regression models for demographic and clinical factors affecting the Wuhan spike-specific immune response after the third dose of COVID-19 vaccination.

Variables	Spike-induced IFN-γ			Spike-induced IL-2			Spike-induced TNF-α			Spike-induced IP-10		
	Coefficient	95% CI	P	Coefficient	95% CI	P	Coefficient	95% CI	P	Coefficient	95% CI	P
Univariable results												
Age, years	-8	-22; 6	0.234	-13	-22; -3	**0.010**	0	-2; 2	0.930	-153	-447; 142	0.298
Gender, male	471	198; 745	**0.001**	279	33; 525	**0.027**	47	26; 69	**<0.001**	4068	-1396; 9532	0.139
Presence of comorbidities	107	-368; 582	0.647	156	-202; 514	0.379	-1	-50; 47	0.956	-1050	-8315; 6215	0.770
BMI	-9	-52; 34	0.668	-17	-47; 12	0.237	-1	-5; 4	0.767	-447	-988; 94	0.102
MS duration, years	-5	-20; 10	0.474	-9	-20; 3	0.130	0	-1; 1	0.891	-180	-412; 53	0.124
MS treatment: Fingolimod	-368	-559; -177	**<0.001**	-308	-394; -222	**<0.001**	-17	-42; 8	0.177	-5565	-8630; -2499	**0.001**
MS treatment duration, years	-16	-45; 13	0.269	-26	-51; -2	**0.036**	0	-3; 3	0.911	-94	-597; 410	0.706
EDSS score ≥ 3	-147	-439; 146	0.313	-242	-445; -39	**0.021**	-1	-28; 26	0.921	-2338	-8962; 4286	0.476
Lymphocyte count × 10^3^/µL	104	9; 199	**0.034**	122	32; 213	**0.010**	-2	-15; 11	0.751	878	-941; 2697	0.332
MS phenotype, Primary-progressive (PP)	347	-109; 802	0.130	203	-136; 541	0.231	46	-14; 106	0.131	3456	-2454; 9367	0.241
Time elapsed from third dose and sample	6	-10; 22	0.468	6	-6; 17	0.305	0	-1; 2	0.594	-39	-266; 188	0.729
Variables in the model after stepwise regression*												
Gender, male	371	131; 609	**0.003**	-----	-----	-----	34	9; 59	**0.010**	-----	-----	-----
MS treatment: Fingolimod	-190	-391; 10	**0.062**	-300	-372; -226	**<0.001**	-----	-----	-----	-5415	-7836; -2996	**<0.001**
Time elapsed from third dose and sample collection	5	-4; 13	0.256	-2	-5; 2	0.373	0	-1; 1	0.744	-72	-189; 44	0.212

PwMS, patients with multiple sclerosis; BMI, body mass index; EDSS, Expanded Disability Status Scale; CI, confidence interval; IFN, interferon; IL, interleukin; TNF, tumor necrosis factor; IP-10, interferon-gamma-induced protein 10. In bold are reported the significant values. * Variables in the model were adjusted for time elapsed from third dose and sample collection.

To assess the immune competence of the enrolled subjects, we analyzed the cytokine production in response to SEB, a non-specific stimulus used as a positive control (**Figures 2C, D**). All HCWs and PwMS demonstrated a robust response to SEB, thus confirming their preserved immune functionality (**Figure 2C**). However, significant differences were observed in the magnitude of IFN-γ, IL-2, and IP-10 production, thus indicating a dysregulated cytokine response in PwMS compared to HCWs. Particularly, PwMS showed a 2-fold reduction in IFN-γ (HCWs median: 6212 pg/mL, IQR: 2995–11663 vs. PwMS median: 2704 pg/mL, IQR: 2001-6830, p=0.011) and IL-2 production (HCWs median: 8433 pg/mL, IQR: 2648–12138 vs. PwMS median: 4050 pg/mL, IQR: 928-7904, p=0.014), while higher IP-10 levels were observed in PwMS than in HCWs (approximately 3-fold increase, p<0.0001). Consistent with the results of the spike-specific response, patients receiving fingolimod showed a more pronounced impairment of the immune response to SEB compared to HCWs, particularly regarding IFN-γ and IL-2 production (p=0.048 and p=0.0001). Nonetheless, the extent of impairment in fingolimod-treated patients is markedly lower than that observed in response to Wuhan spike peptides (IFN-γ SEB: 2.5-fold decrease vs. IFN-γ Wuhan spike: 300-fold decrease; IL-2 SEB: 9-fold decrease vs. IL-2 Wuhan spike: 500-fold decrease) (**Figure 2D**).

### 3.3 Profile of cytokines induced by Delta variant peptides of the spike protein

**Figure 3:**
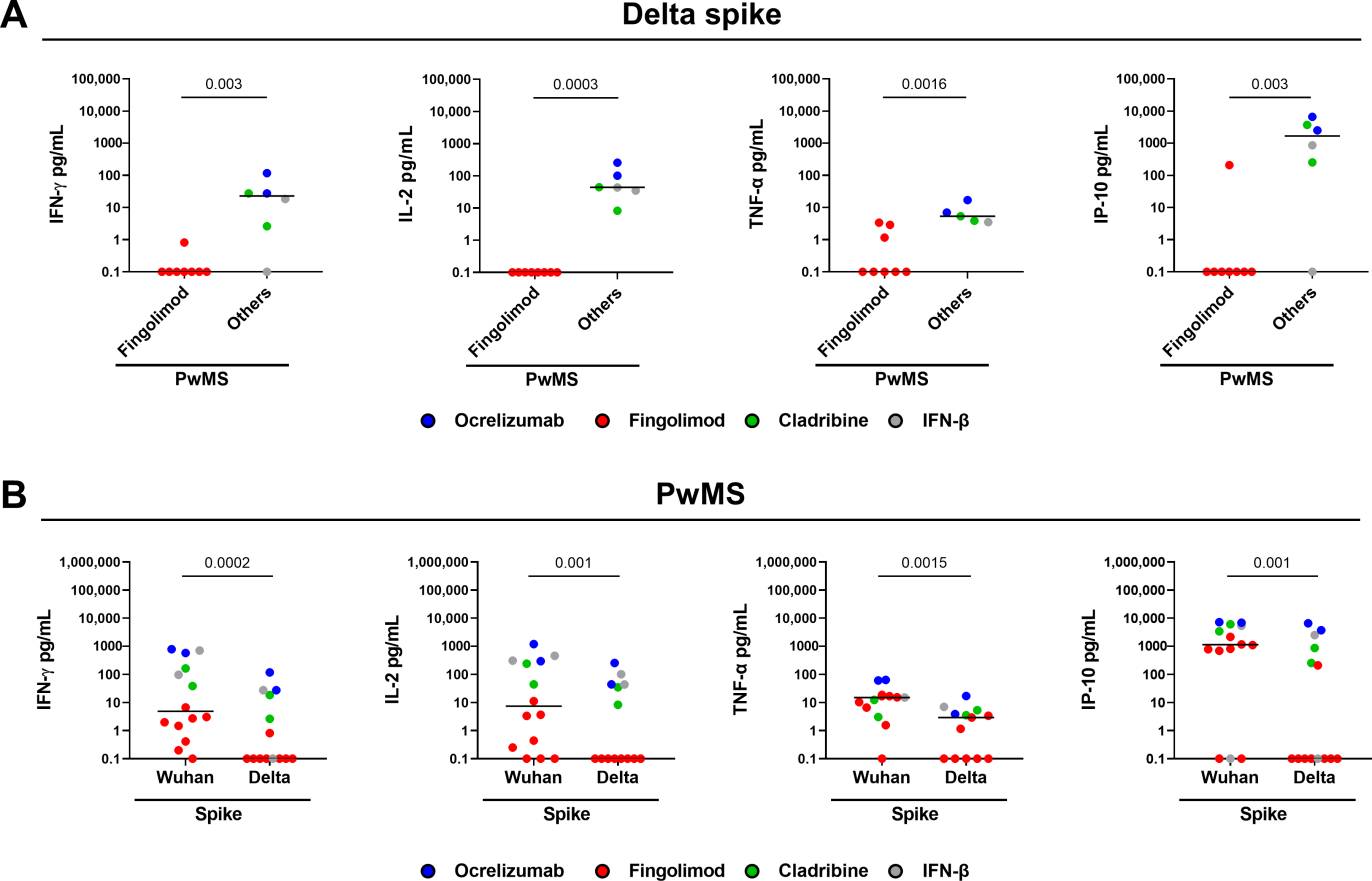
Cytokine/chemokine response to SARS-CoV-2 Delta variant of the spike peptides in PwMS. **(A)** PwMS (n=14) were stratified into two groups: fingolimod (n=8) and others, which includes PwMS treated with ocrelizumab (n=2), cladribine (n=2) and IFN-β (n=2). **(B)** Comparison between the T-cell response induced by the Wuhan spike peptides and that induced by the Delta variant. IFN-γ, IL-2, TNF-α and IP-10 concentrations were expressed in pg/mL with median values indicated by black lines. For the statistical analysis, the Mann-Whitney U test **(A)** and Wilcoxon signed-rank test **(B)** were used and p values < 0.05 were considered significant. PwMS, patients with multiple sclerosis; IFN, interferon; IL, interleukin; TNF, tumor necrosis factor; IP-10, interferon gamma-induced protein 10.

To investigate whether COVID-19 vaccination elicited an immune response against SARS-CoV-2 variants of concern, we evaluated cytokine production after stimulation with peptides derived from the SARS-CoV-2 Delta variant (B.1.617.2) in a subgroup of PwMS (n=14). As shown in **Figure 3A**, the T-cell response to Delta variant significantly differed among DMTs. Specifically, most PwMS treated with ocrelizumab, cladribine, or IFN-β mounted a T-cell response to Delta spike with production of both Th1 cytokines and IP-10. On the other hand, patients treated with fingolimod showed a markedly reduced response compared to other DMTs (IFN-γ: p=0.003; IL-2: p=0.0003; TNF-α, p=0.0016; IP-10, p=0.003). There is no response to Delta spike peptides in fingolimod-treated patients; only one to IFN-γ, three to TNF-α, and one to IP-10. Compared to the response induced by the Wuhan spike peptides, the magnitude of the T-cell response to the Delta variant peptides was significantly lower (IFN-γ: p=0.0002; IL-2: p=0.001; TNF-α, p=0.0015; IP-10, p=0.001) (**Figure 3B**).

### 3.4 Correlations between immunological parameters

**Figure 4:**
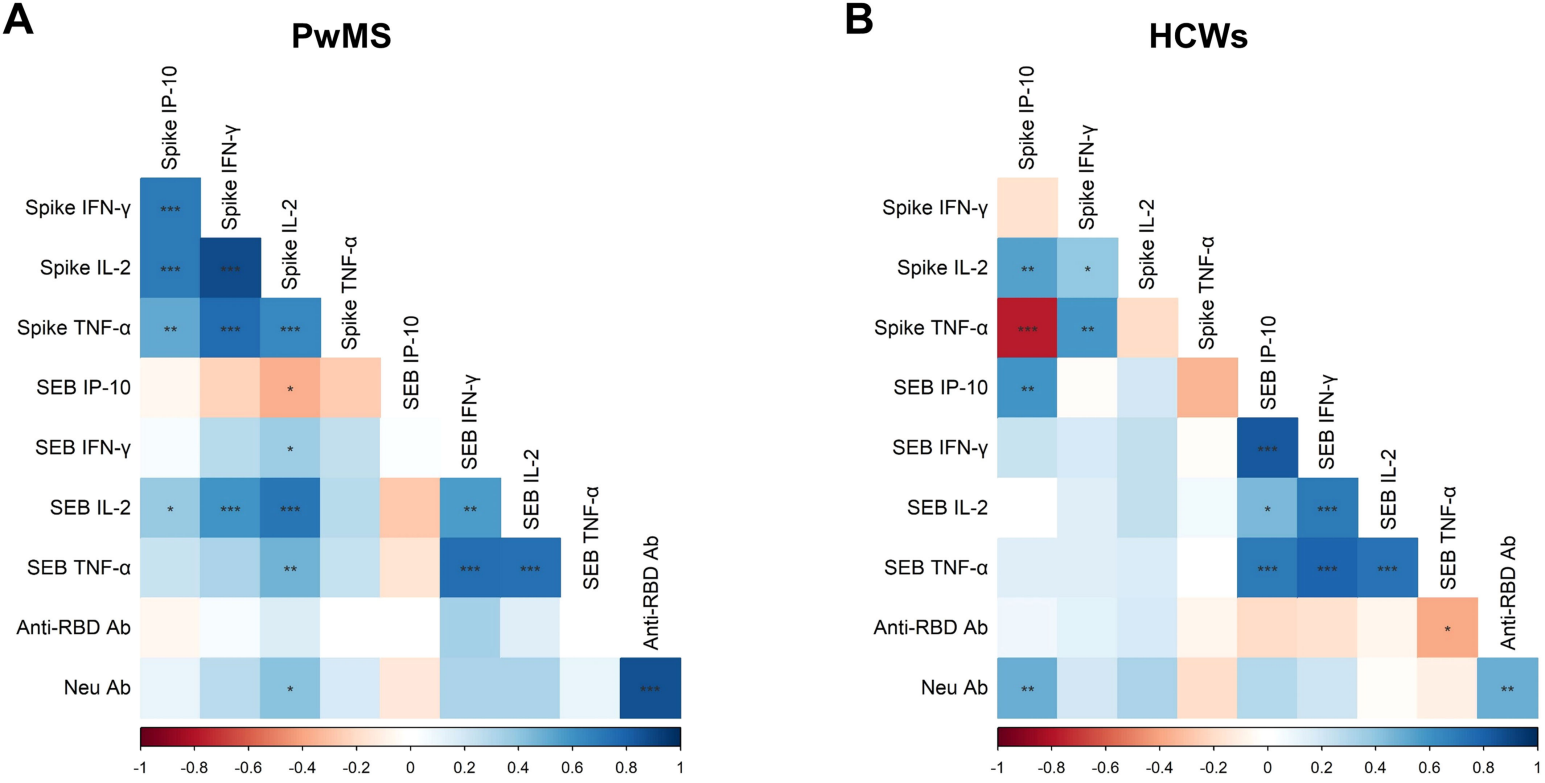
Correlations among the immunological parameters. Correlation matrices in PwMS **(A)** and HCWs **(B)** include the following variables: cytokine/chemokine (IFN-γ, IL-2, TNF-α and IP-10) response to SARS-CoV-2 Wuhan spike peptides and SEB, as well as antibody response measured as anti-RBD and neutralizing antibodies. For the analysis, the non-parametric Spearman’s rank test was performed. Positive (blue) and negative (red) correlations are indicated according to the colour-grade scale, and the colour intensity depends on the strength of the correlation coefficient (ρ). The significance level threshold used is the following: *p<0.05, **p<0.01, ***p<0.001.

A coordinated cytokine response was observed in both cohorts. In PwMS, spike-specific IFN-γ production showed strong positive correlations with IP-10, IL-2, and TNF-α-specific responses (ρ=0.709, p<0.001; ρ=0.882, p<0.001; ρ=0.764, p<0.001, respectively). Moreover, IL-2-spike-specific response positively correlated with both IP-10 and TNF-α (ρ=0.708; p<0.001; ρ=0.650, p<0.001, respectively), and TNF-α-spike-specific production correlated with IP-10 levels (ρ=0.515, p<0.01) (**Figure 4A**). Spike-specific responses in PwMS also correlated with those elicited by SEB stimulation. Particularly, IL-2-spike-specific response was positively associated with SEB-induced IFN-γ, IL-2, and TNF-α levels (ρ=0.367, p<0.05; ρ=0.730, p<0.001; ρ=0.472, p<0.01, respectively), but negatively correlated with SEB-induced IP-10 levels (ρ=-0.370, p<0.05). Moreover, positive correlations were observed between spike-specific IFN-γ and IP-10 productions and SEB-induced IL-2 levels (ρ=0.598, p<0.001; ρ=0.389, p<0.05). These correlations likely emerged within the PwMS cohort due to the greater heterogeneity in lymphocyte counts, attributable to the administration of DMTs, which affect the magnitude of the specific T-cell response.

Interestingly, neutralizing antibody titers, in addition to correlating with anti-RBD antibody levels (ρ=0.870, p<0.001), also showed a positive association with IL-2 production in response to Wuhan spike peptides (ρ=0.415, p<0.05).

In HCWs, cytokine responses elicited by SEB stimulation positively correlated with each other (**Figure 4B**). As for the Wuhan-spike specific response, positive correlations were observed between IFN-γ production and both IL-2 and TNF-α levels (ρ=0.390, p<0.05; ρ=0.590, p<0.01), and between spike-specific IP-10 response and IL-2 levels (ρ=0.549, p<0.01). In contrast, spike-specific IP-10 production negatively correlated with TNF-α levels (ρ=-0.771, p<0.001). Unlike PwMS, in HCWs no significant correlations were observed between spike-specific responses and those induced by SEB stimulation, except for IP-10 levels (ρ=0.594, p<0.01). Furthermore, there was a significant positive correlation between neutralizing antibody titers and spike-induced IP-10 levels (ρ = 0.491, p < 0.01).

### 3.5 SARS-CoV-2 breakthrough infection and immune response

Within the PwMS cohort, 12 patients (38.7%) experienced SARS-CoV-2 breakthrough infections (BIs) during the study period, with a median time of 135 days from administration of the third vaccine dose to BI onset (**Table 1**). Most PwMS (83.4%) developed mild COVID-19, with a median time to nasopharyngeal swab negativization of 13 days (IQR; 10-15), and were treated with antiviral agents or monoclonal antibodies. Among the BI, 50% (6/12) occurred in ocrelizumab-treated patients, 33.3% (4/12) in those receiving fingolimod, and the remaining 16.7% (2/12) in patients treated with IFN-β and cladribine (**Table 4**).

**Table 4:** Demographic, clinical, and immunological factors affecting the incidence of SARS-CoV-2 breakthrough infection in PwMS after the third dose of COVID-19 vaccination.

Patient’s characteristics		SARS-CoV-2 infection		Total	Poisson regression	
		No BI (n=19, 61.3%)	BI (n=12, 38.7%)	(n=31; 100%)	IRR (95% CI)	P
Age in years, median (IQR)		49 (37-56)	50 (45-55)	49 (45-55)	1.05 (0.61-1.80)*	0.859
	23-49	10 (52.6)	6 (50.0)	16 (51.6)	Ref.	
	50-66	9 (47.4)	6 (50.0)	15 (48.4)	0.98 (0.32-3.04)	0.971
Gender, n (%)	Female	17 (89.5)	7 (58.3)	24 (77.4)	Ref.	
	Male	2 (10.5)	5 (41.7)	7 (22.6)	4.05 (1.29-12.76)	**0.017**
Presence of comorbidities, n (%)	No	14 (73.7)	12 (100)	26 (83.9)	–	
	Yes	5 (26.3)	0 (0)	5 (16.1)	-	
BMI (kg/m^2^), median (IQR)		24 (20-27)	24 (22-25)	24 (21-26)	1.02 (0.89 – 1.16)	0.816
	≤ 23.7	10 (52.6)	7 (58.3)	17 (54.8)	Ref.	
	> 23.7	9 (47.4)	5 (41.7)	14 (45.2)	0.78 (0.25-2.44)	0.664
MS duration in years, median (IQR)		16 (8-22)	14 (5-23)	16 (6-22)	1.00 (0.94-1.05)	0.942
	≤ 16 years	9 (47.4)	6 (50.0)	15 (48.4)	Ref.	
	> 16 years	10 (52.6)	6 (50.0)	16 (51.6)	1.03 (0.33-3.19)	0.961
MS treatment, n (%)	Fingolimod	9 (47.4)	4 (33.3)	13 (41.9)	Ref.	
	Cladribine/IFN-β	6 (31.6)	2 (16.7)	8 (25.8)	0.7 (0.13-3.83)	0.682
	Ocrelizumab	4 (21.1)	6 (50.0)	10 (32.3)	2.6 (0.73-9.22)	0.139
MS treatment duration, median (IQR)		7 (2-9)	3 (2-8)	5 (2-8)	0.90 (0.76-1.06)	0.195
	≤ 5 years	8 (42.1)	8 (66.7)	16 (51.6)	Ref.	
	> 5 years	11 (57.9)	4 (33.3)	15 (48.4)	0.45 (0.14-1.50)	0.193
EDSS score, n (%)	<3	7 (36.8)	8 (66.7)	15 (48.4)	Ref.	
	≥3	12 (63.2)	4 (33.3)	16 (51.6)	0.34 (0.10-1.12)	0.076
MS phenotype, n (%)	Primary-progressive (PP)	1 (5.3)	3 (25.0)	4 (12.9)	3.65 (0.99-13.49)	0.052
	Relapsing-Remitting (RR)	18 (94.7)	9 (75.0)	27 (87.1)	Ref.	
Lymphocyte count × 10^3^/µL, median (IQR)		1.28 (0.70-1.51)	1.28 (0.73-1.56)	1.28 (0.70-1.51)	0.87 (0.52-1.47)	0.610
	Linf _p50 = 0	9 (52.9)	5 (45.5)	14 (50.0)	Ref.	
	Linf_p50 = 1	8 (47.1)	6 (54.5)	14 (50.0)	1.18 (0.36-3.87)	0.784
Immune response						
Anti-RBD Ab, median (IQR)		249 (16-4895)	50 (0.7-314)	(0.8-1027)	1.00 (1.00-1.00)	0.167
Anti-RBD Ab score, n (%)	< 7.1 BAU/mL	3 (15.8)	6 (50.0)	9 (29.0)	Ref.	
	≥7.1 BAU/mL	16 (84.2)	6 (50.0)	22 (71.0)	0.27 (0.09-0.83)	**0.022**
Spike IFN-γ, median (IQR)		39 (2-385)	127 (2-533)	86 (1-476)	1.00 (1.00-1.00)	0.545
Spike IL-2, median (IQR)		32 (3-398)	227 (2-310)	81 (3-325)	1.00 (1.00-1.00)	0.442
Spike TNFα, median (IQR)		15 (4-60)	17 (5-28)	15 (4-39)	0.99 (0.98-1.01)	0.373
Spike IP-10, median (IQR)		3425 (817-8704)	6215 (933-7032)	4347 (817-7183)	1.00 (1.00-1.00)	0.620

BI, breakthrough infection; PwMS, patients with multiple sclerosis; IQR, interquartile range; BMI, body mass index; EDSS, Expanded Disability Status Scale; RBD, receptor-binding domain; Ab, antibodies; IFN, interferon; IL, interleukin; TNF, tumor necrosis factor; IP-10, interferon-gamma-induced protein 10. IRR: incidence rate ratio estimated with Poisson regression; CI: confidence interval; n, Number; Ref: reference category. *For 10-year increment. In bold are reported the significant values.

Interestingly, among the demographic and clinical factors, Poisson regression analysis identified sex as a significant factor associated with the risk of BI in PwMS. Male sex was significantly associated with a higher incidence rate of BI compared to female sex (IRR: 4.05, 95%CI:1.29-12.76, p=0.017). Moreover, PwMS with a primary progressive disease showed a higher incidence rate of BI (IRR: 3.65, 95%CI: 0.99-13.49, p=0.052) compared to those with relapsing-remitting MS.

Moreover, we evaluated whether the cell-mediated immunity induced after the third vaccine dose influenced the risk of subsequent SARS-CoV-2 infections in PwMS. As shown in **Supplementary Figure 1** and supported by the Poisson regression analysis (**Table 4**), the spike-specific response was not associated with increased protection against BI. In contrast, a significant association was observed between antibody response and a reduced risk of BI (IRR: 0.27, IQR: 0.09–0.83, p = 0.022) (**Table 4**). This association explains the higher susceptibility of ocrelizumab-treated patients to SARS-CoV-2 infection, as ocrelizumab treatment significantly impairs the antibody response, as shown in previous studies (13, 23). By contrast, most fingolimod-treated patients induced an antibody response, although its magnitude was lower compared to that observed in healthy controls, while cladribine and IFN-β did not significantly affect the humoral response.

## 4 Discussion

This prospective study provides an in-depth immunological characterization of the spike-specific response following the third dose of COVID-19 vaccination in PwMS undergoing different DMTs by evaluating soluble biomarkers beyond IFN-γ, including IL-2, TNF-α and IP-10, as surrogate markers of the cell-mediated response. Moreover, the immune response was compared with that of a cohort of HCWs matched for age and sex, and the potential association between these immune factors and the risk of BI was investigated.

To date, most studies have focused on the evaluation of the spike-specific T-cell response, selectively assessing IFN-γ response (13, 18, 32, 36, 44). The identification of additional immunological biomarkers may be important to gain a more comprehensive view of the cell-mediated response induced by COVID-19 vaccination. Data on the vaccine-induced response, evaluated as production of both Th1 cytokines (IFN-γ, IL-2, TNF-α) as well as IP-10, are scarce (48, 54). Few studies have examined the functional spike-specific CD4^+^ and CD8^+^ T cell responses by evaluating IFN-γ-, IL-2, or TNF-α-producing T cells by flow cytometry in PwMS after the third dose of COVID-19 mRNA-vaccines (22, 45–47). In this study, we employed a user-friendly whole-blood assay to measure Th1 cytokines and IP-10, serving as surrogate markers for the cell-mediated immune response following COVID-19 vaccination.

We demonstrated that PwMS receiving DMTs have an immune system capable of responding to a nonspecific stimulus such as SEB. However, their cytokine response appears dysregulated compared to that observed in HCWs. This difference becomes even more significant when evaluating antigen-specific immune responses, such as those elicited in response to SARS-CoV-2 spike antigens after COVID-19 vaccination. In this case, most PwMS mounted a T-cell response by releasing Th1 cytokines (IFN-γ, IL-2, TNF-α) as well as IP-10. This data aligns with a recent study showing that the immune response to SARS-CoV-2 vaccines in PwMS is unbalanced towards a Th1 phenotype, predominantly characterized by IL-2 and IFN-γ (48).

Nonetheless, the magnitude of the Th1 response to the Wuhan spike peptides was significantly lower than that observed in HCWs, with IFN-γ and TNF-α levels reduced by six-fold, and IL-2 levels decreased by approximately three-fold. Notably, significant variations in the immune response were observed according to the ongoing DMTs, with fingolimod-treated patients presenting the most immunocompromised response, especially in terms of both IFN-γ and IL-2 production. These results confirm previous studies showing reduced IFN-γ levels (13, 48) or frequencies of IFN-γ and IL-2-producing T cells in fingolimod-treated patients (22, 46).

These findings are consistent with the fingolimod’s mechanism of action. Fingolimod acts as a sphingosine 1-phosphate (S1P) receptor modulator and hinders lymphocyte egress from lymph nodes, thereby leading to a reduced lymphocyte count in the peripheral blood (3) and a diminished capacity to mount an effective immune response (55). In addition to MS treatment, male sex was identified as an independent factor influencing the spike-specific immune response, particularly affecting TNF-α production.

Despite widespread vaccination efforts, the emergence of SARS-CoV-2 VOCs has reduced the protective efficacy of existing COVID-19 vaccines (56). However, whereas it has been shown that the antibody responses against VOCs are markedly reduced, because these variants have acquired the ability to evade the antibody recognition (57, 58), the T-cell response appears to be more heterogeneous (59, 60). Consistent with earlier findings (59, 61), we found that COVID-19 vaccines based on the original Wuhan spike protein induce T-cell responses that also cross-react with the Delta variant, as shown by various immunological biomarkers. In this context, PwMS generated a Delta spike-specific response by producing Th1 cytokines and IP-10, though certain DMTs, such as fingolimod, may impair this response, confirming results generated with the Wuhan spike antigen.

Although the cross-reactivity was maintained, the magnitude of the T-cell response to the Delta variant peptides in PwMS was significantly lower than that induced by the Wuhan spike peptides. This result is expected, considering that the antigen used in the first mRNA vaccines was based on the spike protein derived from the original Wuhan strain.

In the PwMS cohort, and to a lesser extent in HCWs, spike-specific cytokine levels showed positive correlations with one another, indicating that the immunological parameters analyzed reflect immune responsiveness. Interestingly, in addition to the already known correlation with anti-RBD antibodies (20, 23), we demonstrated a positive association between neutralizing antibody titers and the levels of IL-2 in PwMS. Altogether, this evidence supports the use of IL-2 as a valuable biomarker for assessing both the cell-mediated and antibody response to COVID-19 vaccination in PwMS.

IL-2, as well as IFN-γ and TNF-α, are cytokines mainly produced by Th1 lymphocytes. IFN-γ contributes to macrophage activation and controls the differentiation of naïve CD4^+^ T cells into Th1 effectors, which in turn mediate cellular immunity against viral and intracellular bacterial infections (41). TNF-α is involved in various processes, including cell survival, cell death, inflammation, and immune cell activation (42). IL-2 indirectly favors antibody response by promoting T-cell activation and proliferation as well as the differentiation of T follicular helper cells, which are important for B cell maturation (62). Moreover, IL-2 contributes to the generation of plasma cells responsible for antibody production (63, 64).

In HCWs, the neutralizing titer positively correlated with the levels of IP-10. The latter is a chemokine, induced by IFN-γ, that promotes the chemotaxis of activated T and B lymphocytes to the sites of inflammation (65, 66). Moreover, IP-10 drives activated B cells to differentiate into plasma cells (67).

Regarding the incidence of BI, 38.7% (12/31) of PwMS in our cohort experienced SARS-CoV-2 infection after three doses of COVID-19 vaccines. Most of them reported a mild COVID-19, likely explained also by the lower pathogenicity of the Omicron variant (68, 69), which was the predominant variant circulating in Italy during the follow-up period of this present study (70).

Among the demographic and clinical factors analyzed, sex emerged as a significant variable associated with the risk of BI in PwMS. Specifically, male patients exhibited a 4-fold increased risk of BI compared to females. This finding is consistent with previously reported sex-related differences in vaccine-induced response, wherein females mount a more robust immune compared to males (71, 72). Moreover, the relapsing-remitting phenotype of MS disease was associated with a greater protection compared to the primary progressive form, thus confirming previous data (38, 73).

Among DMTs, patients receiving ocrelizumab showed the highest incidence rate of BI, followed by those treated with fingolimod and, finally, patients with cladribine/IFN-β. These results are consistent with data from other studies, identifying patients on ocrelizumab and fingolimod as those at greatest risk of BI (74–77).

.Furthermore, this finding is consistent with the known mechanism of action of ocrelizumab, which acts as CD20-depleting B cell agent (3), underscoring the pivotal role of the antibody response (78). In our PwMS cohort, we confirmed that mounting an antibody response confers protection against infection with an estimated 70% reduction in BI risk, as previously demonstrated (38). To note, SARS-CoV-2 infection occurred approximately 5 months after the administration of the third dose, a time frame that corresponds to the decline of the vaccine-induced antibody response, as widely reported in vary longitudinal studies (13–16).

Unlike the antibody response (79), we did not find any association between the spike-specific cell-mediated response and the protection against SARS-CoV-2 infection for any of the soluble factors evaluated. However, although the lack of a proper T-cell response does not imply an increased BI risk, we have previously showed that a reduced IFN-γ T-cell response adversely affects the time to have a swab negativization by increasing the time required to achieve viral clearance (38). Indeed, fingolimod-treated patients require approximately 7 additional days to test negative. A prolonged persistence of the virus may promote its replication, increasing the likelihood of the emergence of new variants in the individual and spread the infection to the community (17).

Some limitations of the study are acknowledged. Firstly, the small sample size may have limited the ability to perform more in-depth analyses; however, the cohort was thoroughly characterized both immunologically and clinically. Secondly, among the SARS-CoV-2 VOC, only the immune response to Delta variant was evaluated, and a direct comparison with the corresponding response in HCWs is lacking.

On the other hand, a major strength of this study is the comprehensive characterization of the spike-specific immune response. In addition to assessing the IFN-γ production, this study also measured IL-2, TNF-α and IP-10 levels, thus providing a broader overview of the T-cell functionality and cytokine response. Moreover, as far as we know, this is the first study evaluating the association between these immunological biomarkers and the risk of BI. In addition, our immunological data were obtained using a friendly-to-use whole blood standardized method that has been thoroughly validated in previous studies in COVID-19 (51, 80) or in other diseases as tuberculosis (81). However, we cannot rule out that advanced techniques directly measuring the T-cell responses, such as intracellular cytokine staining, rather than using surrogate markers, may provide different results. To our knowledge, a recent flow cytometry study corroborated our findings by demonstrating lower percentages of responding and triple-positive (IFN-γ, IL-2, TNF-α) T cells in PwMS compared to healthy controls, particularly within the depleting/sequestering-out subgroup, such as patients treated with fingolimod (45). Unfortunately, the study did not examine the association between the T-cell response and the risk of SARS-CoV-2 breakthrough infections.

As a clinical implication of our findings, this study contributes to refining the stratification of SARS-CoV-2 infection risk in PwMS treated with DMTs, particularly highlighting patients treated with ocrelizumab and fingolimod as higher-risk groups. These results support the consideration of tailored vaccination approaches, including adjusted booster timing or additional prophylactic interventions. While our data focus on specific DMTs, this framework may be extended to other DMTs and warrants further investigation. Among the immunological biomarkers analyzed, IL-2 emerged as a promising complementary marker of vaccine-induced immunity, showing correlations with both humoral and cell-mediated responses. Nevertheless, additional studies are necessary to confirm its clinical utility and establish thresholds.

In conclusion, this study provides evidence of the spike-specific cytokine and chemokine response in PwMS undergoing DMTs following the third dose of COVID-19 vaccination. Our findings showed that PwMS mount a Th1-type immune response, broadly resembling that observed in HCWs, albeit with significantly reduced levels of IFN-γ and IL-2. Notably, this impaired Th1 response is not associated with a risk of SARS-CoV-2 infection. Importantly, our results identify immunological biomarkers beyond IFN-γ, particularly IL-2, as additional tools to assess the cell-mediated immune response to COVID-19 vaccination. The relevance of these immunological biomarkers may extend beyond COVID-19, for offering insight into host immune responses to other infectious agents and vaccinations.

## Data Availability

The raw data are available in our institutional repository (rawdata.inmi.it), subject to registration. The data can be found by selecting the article of interest from a list of articles ordered by the year of publication. No charge for granting access to data is required. In the event of a malfunction of the application, the request can be sent directly by e-mail to the library (biblioteca@inmi.it).
