# Supplementary material for: Minimal association between Th1-specific responses to COVID-19 vaccines and SARS-CoV-2 breakthrough infections in multiple sclerosis patients receiving disease-modifying therapies
Source: Front Immunol. 2025 Oct 30;16:1682049. doi: 10.3389/fimmu.2025.1682049 (PMC12611814; doi:10.3389/fimmu.2025.1682049)
Supplement: Supplementary file 1 [file DataSheet1.docx]

Supplementary Figures


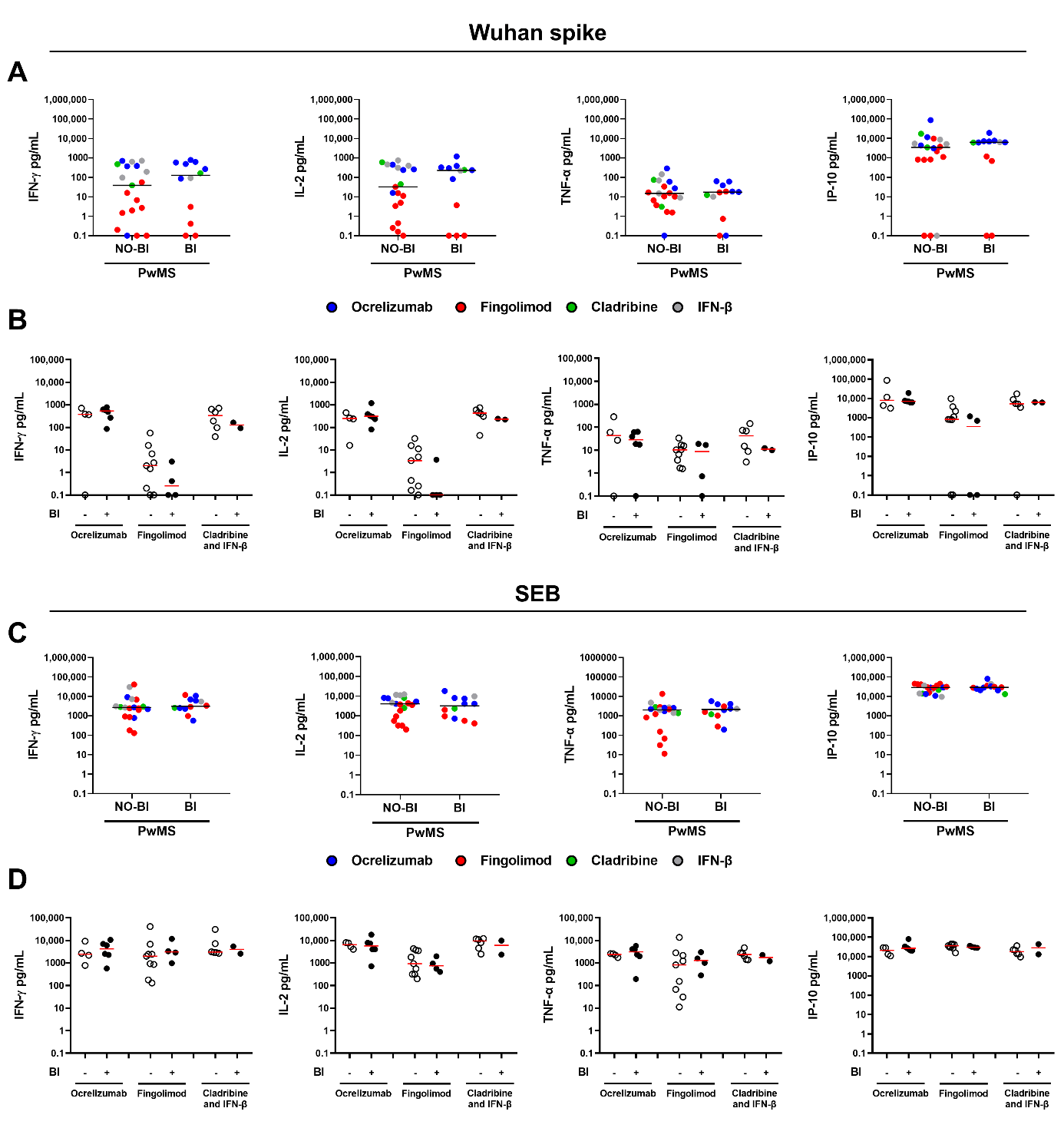


**Supplementary Figure 1.** Cytokine/chemokine response to SARS-CoV-2 Wuhan spike peptides and SEB in PwMS after the third vaccine dose stratified according to the subsequent occurrence of SARS-CoV-2 breakthrough infection (BI). (A, C) PwMS were divided into two groups: BI group (n=12) consisting of subjects who had SARS-CoV-2 infection following the third vaccine dose, and NO-BI group (n=19) including subjects who did not become infected during the follow-up period. Each coloured dot corresponds to a specific DMT as indicated in the legend. (B, D) PwMS were also categorized by the ongoing DMT. IFN-γ, IL-2, TNF-α and IP-10 concentrations were expressed in pg/mL with median values indicated by red lines. For the statistical analysis, the Mann-Whitney U test was used and p values < 0.05 were considered significant. Abbreviations: PwMS, patients with multiple sclerosis; DMT, disease-modifying treatment; SEB, Staphylococcal enterotoxin B; IFN, interferon; IL, interleukin; TNF, tumor necrosis factor; IP-10, interferon gamma-induced protein 10.
